# Biological and practical implications of genome-wide association study of schizophrenia using Bayesian variable selection

**DOI:** 10.1038/s41537-019-0088-6

**Published:** 2019-11-19

**Authors:** Benazir Rowe, Xiangning Chen, Zuoheng Wang, Jingchun Chen, Amei Amei

**Affiliations:** 1 Department of Mathematical Sciences, University of Nevada, Las Vegas, NV USA; 2 Nevada Institute of Personalized Medicine, University of Nevada, Las Vegas, NV USA; 3 Department of Biostatistics, Yale School of Public Health, New Haven, CT USA; 4 Present Address: 410 AI, LLC, Germantown, MD USA

**Keywords:** Schizophrenia, Biomarkers

## Abstract

Genome-wide association studies (GWAS) have identified over 100 loci associated with schizophrenia. Most of these studies test genetic variants for association one at a time. In this study, we performed GWAS of the molecular genetics of schizophrenia (MGS) dataset with 5334 subjects using multivariate Bayesian variable selection (BVS) method Posterior Inference via Model Averaging and Subset Selection (piMASS) and compared our results with the previous univariate analysis of the MGS dataset. We showed that piMASS can improve the power of detecting schizophrenia-associated SNPs, potentially leading to new discoveries from existing data without increasing the sample size. We tested SNPs in groups to allow for local additive effects and used permutation test to determine statistical significance in order to compare our results with univariate method. The previous univariate analysis of the MGS dataset revealed no genome-wide significant loci. Using the same dataset, we identified a single region that exceeded the genome-wide significance. The result was replicated using an independent Swedish Schizophrenia Case–Control Study (SSCCS) dataset. Based on the SZGR 2.0 database we found 63 SNPs from the best performing regions that are mapped to 27 genes known to be associated with schizophrenia. Overall, we demonstrated that piMASS could discover association signals that otherwise would need a much larger sample size. Our study has important implication that reanalyzing published datasets with BVS methods like piMASS might have more power to discover new risk variants for many diseases without new sample collection, ascertainment, and genotyping.

## Introduction

Schizophrenia is a severe psychiatric disorder with an estimated global lifetime prevalence of 0.4−0.75% with no significant differences across urban, rural, and mixed sites or genders.^1,2^ While being a low prevalence disorder, it has substantial societal burden.^3^ The estimated heritability of schizophrenia ranges from 70 to 90%.^4^ The common susceptibility variants of such disease are typically identified by association studies, such as genome-wide association studies (GWAS). In these studies, single nucleotide polymorphisms (SNPs) are often tested one at a time. In recent years, genetic studies of schizophrenia have made substantial progress. Since the report of the major histocompatibility complex (MHC) locus on chromosome 6 in 2009,^5^ the number of schizophrenia-associated genetic loci has risen to 5 loci in 2011^6^ and to 108 loci in 2014.^7^ This increase in the number of significant loci could be partially explained by the increase in the sample size of the studies that led to improvement in the statistical power of the association tests. However, these studies also suggest that common variants usually have small to medium effects that makes them hard to reach the typical GWAS significance threshold (*P* = 5 × 10^−8^). The application of regression methods on set of genetic variants with appropriate prior specification may have the potential to uncover the largely hidden heritability.

It is well known that the single-SNP approach has its advantage in its simplicity of use, well-established pipeline and low computational burden. However, one of the major drawbacks is that it may miss some potential additive effects derived from sets of SNPs or genes. Methods like Bayesian variable selection (BVS) take these considerations into account and analyze multiple loci simultaneously. For diseases with complex genetic architecture, such as schizophrenia, it is possible that BVS combined with powerful computing resources might be superior to single-SNP approach. Indeed, Bayesian methods have demonstrated their abilities in search for genetic risk factors in schizophrenia and other complex disorders.^8–10^ Since then, much have been developed in the area of Bayesian GWAS.^11,12^ The Posterior Inference via Model Averaging and Subset Selection (piMASS) algorithm is one of such examples.^13^ It offers a BVS procedure that is designed for continuous phenotypes with an extension to binary phenotypes using a probit link function. By considering a set of genetic variants, piMASS extracts more information beyond the marginal associations in standard single-SNP analyses while maintaining reasonable computation time.^13^ Therefore, piMASS has potential to uncover more associations through reanalysis of existing GWAS datasets. In this study, we chose a dataset with a moderate sample size, molecular genetics of schizophrenia (MGS) that has previously been analyzed using univariate methods^5^ and reanalyzed the dataset using piMASS. We hypothesize that piMASS can discover more associations when applied to MGS dataset compared with the single-SNP methodology used in ref. ^5^ Specifically, we use piMASS to evaluate associations of a set of genetic variants in a moderate sample size of 5334 subjects (2681 cases and 2653 controls) with binary phenotype using posterior inclusion probabilities (PIPs)—measures of confidence that individual variants have nonzero effects, no interaction effects considered. Such direct comparison with one of the most common GWAS methods can shed the light on the utility of piMASS in analysis of moderate size datasets. We used permutation test to validate our findings with an independent schizophrenia case–control dataset of similar size (2895 cases and 3836 controls). Our results indicate that compared with single-SNP approaches, BVS method, such as piMASS, could discover association signals with a relatively small sample size that might have been undetectable by single-SNP approaches.

## Results

In the discovery dataset MGS, region 29 on chromosome 15 containing SNPs between 83,907,801 and 86,887,657 reached genome-wide significance after Bonferroni correction (Rank 1, *P*_disc_ = 1.43 × 10^−5^, *P*_vali_ = 0.001). rs16940789, rs16941261, rs4887364, rs991728, rs2114252, and rs994068 (Supplementary Table 1) are among the SNPs with top 1% highest PIP and mapped to gene NTRK3 in the SchiZophrenia Gene Resource database, SZGR 2.0 (https://bioinfo.uth.edu/SZGR/), a comprehensive database of variants and genes reported to have an association with schizophrenia.^14^ NTRK3 has been shown to be associated with bipolar and other psychiatric disorders.^15–17^ The gene encodes a member of the neurotrophic tyrosine receptor kinase (NTRK) family, which is involved in nervous system. rs16940789 was also mapped to gene LINC00052 (an RNA gene that is affiliated with the noncoding RNA). The locus had not been previously reported in refs ^7,18^

**Fig. 1:**
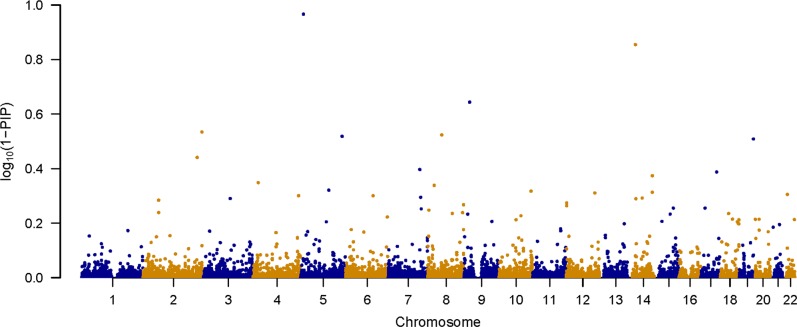
Manhattan plot of 1-PIP for the MGS dataset

Although only one locus surpassed Bonferroni correction (*P*_disc_ < 7.9 × 10^−5^), some regions with the empirical *p* value (*P*_disc_) that are close to the cutoff might still be of interest because Bonferroni correction is known to be too conservative and we used a design of sliding window with overlapping SNPs. Table 1 lists 12 regions with the best association metric (*P*_disc_) based on 100,000 permutations using the MGS dataset, as well as their corresponding empirical *p* values based on 1000 permutations using the Swedish Schizophrenia Case–Control Study (SSCCS) dataset (*P*_vali_). For each of the 12 regions reported in Table 1, we ranked the 1000 SNPs in terms of the PIP generated from the initial run using the MGS dataset and listed the top ten SNPs based on PIP within each region (Supplementary Table 1). The Manhattan plot of the PIP of individual SNP from the initial run using the MGS dataset is shown in Fig. 1 using −log_10_(1-*PIP*) as the *y*-axis.

**Table 1:** Regions with best association metrics (*P*_disc_) based on permutation test

Chr	Region^a^	Start position^b^	End position^b^	Rank^c^	*P*_disc_^d^	*P*_vali_^e^
15	29	83,907,801	86,887,657	1	1.43E−05	0.001
19	5	15,724,023	22,638,628	2	1.67E−04	<0.001
14	33	86,399,092	90,573,122	3	2.20E−04	<0.001
9	24	34,905,605	70,379,322	4	2.50E−04	0.002
14	6	29,288,170	33,177,081	5	3.00E−04	<0.001
8	30	53,113,091	57,376,926	5	3.00E−04	0.001
20	1	9795	2,715,620	7	3.33E−04	<0.001
18	15	28,642,588	33,646,071	8	5.00E−04	<0.001
15	28	80,260,648	85,190,202	9	5.50E−04	<0.001
1	36	81,955,643	85,727,849	10	5.67E−04	<0.001
13	41	98,395,342	101,641,945	11	6.00E−04	<0.001
3	15	22,010,347	25,354,138	12	7.50E−04	0.001

^a^Regions were assigned separately to each chromosome starting from 1

^b^Start position reflects the position of the first SNP included in the region, end position reflects the position of last SNP included in the region

^c^Rank is based on empirical *P* value calculated from permutation test using the MGS dataset

^d^Empirical *P* value based on 100,000 or less permutations using the discovery dataset (MGS)

^e^Empirical *P* value based on 1000 permutations using the validation dataset (SSCCS)

There are 5 SNPs among the 120 SNPs listed in Supplementary Table 1 that have been mapped to genes associated with schizophrenia in the GWAS Catalog^19^ (Table 2). Average C-scores based on Combined Annotation-Dependent Depletion (CADD) method are also listed in Table 2.^20^ SNPs rs993804 and rs4858697, located at 3p24.2, are in Linkage Disequilibrium (LD) (*R*^2^ = 0.45) and are mapped to the gene *AC092422.1 (RARB)* (chr3:24687919-25174305). *AC092422.1 (RARB)* had been reported to be associated with schizophrenia and bipolar disorder in a meta-analysis for genome-wide association data using European–American samples.^21^ SNP rs2044117, located at 13q32.3, is mapped to genes *NALCN-AS1* and *NALCN*. *NALCN-AS1* was reported to be associated with schizophrenia and bipolar disorder.^21^ The *NALCN* was reported to be associated with multiple traits including bipolar disorder, eating disorder, schizophrenia, adolescent idiopathic scoliosis, HIV-associated dementia, psychosis, recurrent major depressive disorder, etc.^22–26^ SNP rs9554752 is also mapped to *NALCN* and it is in LD with rs2044117 (*R*^2^ = 0.11). SNP rs915071, located at 14q12, is mapped to genes *AL352984.2*: *LOC105370439* and *LOC105370440* that were reported to be associated with schizophrenia and bipolar disorder.^21^ The CADD scores for SNPs in Table 2 range from 0.898 to 9.444. Resulting CADD scores point to the fact that piMASS alone cannot discover causal variants. The main reason is that piMASS is a tool for association testing and hence the SNPs discovered are not necessarily causal. Moreover, other study characteristics like region-based design and unimputed dataset add to the fact that additional steps may be necessary to investigate the pinpointed regions for causal SNPs.

**Table 2:** SNPs with their mapped genes

Chr	Gene Region	SNP	Position^a^	MAF^b^	PIP	C-score^c^
3	AC092422.1 (RARB)	rs993804	25,070,680	0.27	0.059	5.917
3	AC092422.1 (RARB)	rs4858697	25,075,091	0.46	0.044	2.97
13	NALCN, NALCN-AS1	rs2044117	101,055,958	0.13	0.124	9.444
13	NALCN	rs9554752	101,073,961	0.35	0.040	1.960
14	LOC105370439, LOC105370440	rs915071	31,964,652	0.40	0.738	0.898

^a^Position is referred to NHGRI-EBI GWAS Catalog

^b^Minor allele frequency (MAF) in the 1000 Genomes Phase 3 combined population

^c^Average C-score based on Combined Annotation–Dependent Depletion (CADD) method

Based on the permutation test on the discovery dataset, region 5 on chromosome 19 has the second smallest empirical *p* value (Rank 2, *P*_disc_ = 1.67 × 10^–4^, *P*_vali_ ≤ 0.001. Among the SNPs having the highest 1% PIPs within this region, there are six SNPs (rs2965189, rs2916074, rs4808200, rs4808203, rs4808964, and rs10419912) and they are in high LD (*R*^2^ ≥ 0.93) with rs2905426 located at 19p13.11. The SNP rs2905426 is a variant belongs to a regulatory region of genes *GATAD2A* and *MAU2*. This variant was previously reported to be associated with schizophrenia from the Psychiatric Genomic Consortium (PGC) study, where 128 independent associations with 108 conservatively defined loci were identified in a GWAS of up to 36,989 cases and 113,075 controls^7^ (see also refs ^27–29^). Based on SZGR 2.0 we found 63 SNPs out of the 120 SNPs that are mapped to 27 genes in the SZGR 2.0 database and shown to be associated with schizophrenia (Supplementary Table 1).

**Fig. 2:**
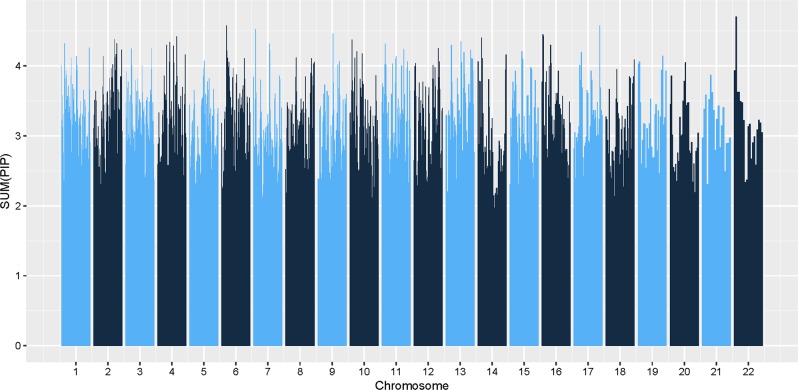
piMASS genome-wide region-based performance of the SSCCS dataset. The sum of posterior inclusion probabilities (PIPs) for each of the 1244 overlapping regions spanning 22 chromosomes of the SSCCS dataset

**Fig. 3:**
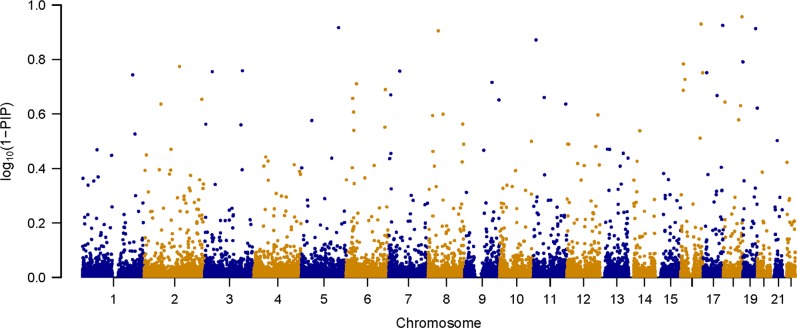
Manhattan plot of 1-PIP for the SSCCS dataset

We also conducted an overlap analysis between MGS and SSCCS datasets based on a single run of piMASS without the permutation test. The results of the piMASS analysis of the SSCCS dataset based on sum of PIPs for each region are presented in Fig. 2. Figure 3 shows Manhattan plot based on individual PIPs using −log_10_(1-*PIP*) as the *y*-axis. The overlap analysis suggests that 46.3% of the pairs were in LD with each other in the 100,000 bp overlap among top 1% of the SNPs of the top 5% of the regions (Supplementary Table 2). Overlap percentages using other choices of distance (in bp) are given in Supplementary Table 3. rs7746199 chr6:27261324 belongs to extended MHC region and has been previously implicated in association with schizophrenia.^5^ rs7746199 as well as rs2747421, rs2535238, rs375984, rs2747421, rs2535238, rs375984 SNPs are in the consensus set and are in LD with rs1153229265 of chr6 reported by PGC^7^ (Supplementary Table 4).

## Discussion

Guan and Stephens developed a BVS regression model to large-scale datasets primarily focusing on analysis of quantitative traits.^13^ In this study, we used the BVS regression model to conduct a case–control GWAS of schizophrenia with binary phenotype for datasets with moderate sample sizes (cases/controls for MGS and SSCCS datasets are 2681/2653 and 2895/3836, respectively). We have demonstrated that the BVS methods can discover association signals that otherwise would need a much larger sample size to discover.

Application of BVS to the MGS European ancestry case–control sample produced 17 regions having *P*_disc_ < 8 × 10^−4^ based on 100,000 permutations. Among them, 12 regions were validated using the SSCCS dataset with *P*_vali_ ≤ 0.002 based on 1000 permutations. The region with the smallest *p* value, which belongs to chromosome 15 reached genome-wide significance. SNPs with the highest PIP from this region are mapping to gene NTRK3 that encodes a member of the NTRK family and has been reported to be associated with bipolar and other psychiatric disorders. Five SNPs among the twelve validated regions are mapped to genes that are known to be associated with schizophrenia and other mental disorders, such as bipolar disorder, eating disorder, adolescent idiopathic scoliosis, psychosis, recurrent major depressive disorder.^21–26^ A cluster of six SNPs on chromosome 19 are found to be in high LD with rs2905426, which is mapped to the regulatory region of *GATAD2A* and *MAU2*, genes that are known to be associated with schizophrenia and bipolar disorder.^7^

Our BVS analysis of the MGS European ancestry case–control dataset identified one region that reached genome-wide significance, while the original GWAS of the MGS case–control dataset that used single-SNP approach did not find any significant signal.^5^ This result indicates that piMASS method has the potential to uncover more associations even in moderate sample size setting, as compared with the single-SNP approach. Our results suggest that BVS methods, such as piMASS, can be used to reanalyze published datasets to discover new risk variants for many diseases without new sample collection, ascertainment, and genotyping.

Our analysis produced a single region that achieved genome-wide significance that has not been reported in large-scale schizophrenia GWAS. The region is on chromosome 15 containing SNPs between 83,907,801 and 86,887,657. Among SNPs with top 1% highest PIP in this region is rs16940789 in the genes LINC00052 and NTRK3.

While we were able to demonstrate the potential superiority of piMASS over standard GWAS, there are a few ways it could be further improved. First, performing genotype imputation for both the discovery and validation datasets could provide more precise comparison between the two datasets. Imputation enables direct comparison between datasets, which could be beneficial to the understanding of the piMASS performance. Second, given that population stratification and cryptic relatedness are among the confounding factors in genetic association studies,^30,31^ a strategy that accounts for population structure could improve the accuracy of association discovery and extend the application to datasets with less homogeneous population structure. Third, comparison of piMASS with other methods beyond single-SNP approach can help placing it in a hierarchy of other GWAS tools for real data. Another potential direction of further research is the extension of the model to handle categorical response data, thus allowing to analyze phenotypes with polychotomous scale such as addiction and other diseases. The classical approach to multinomial response data is to fit a categorical response regression using maximum likelihood and make inference about the model based on the associated asymptotic theory. It has been pointed out that the inference based on the classical approach is questionable for small sample sizes and Bayesian methods provided an attractive alternative.^32^ Having reached the conclusion that it is possible to uncover more associations using single dataset with moderate sample size, it now makes sense to move on to apply piMASS to larger, more heterogeneous datasets, imputed datasets and ultimately perform meta-analysis of the results of the BVS analysis of multiple datasets.

## Methods

### GWAS datasets

In discovery, we performed association analysis using the MGS study (*n* = 5334) that consists of 2681 schizophrenia cases and 2653 healthy controls of European ancestry. Details of the dataset have previously been described.^5^ In validation, we chose batches 5 and 6 of the SSCCS dataset (*n* = 6731), including 2895 cases and 3836 controls.^33^ Both MGS and SSCCS GWAS datasets were downloaded from NIMH Genetic Repository and Resource (https://www.nimhgenetics.org/) upon approval. The genotypes were downloaded from NIMH without further quality check because the genotypes from NIMH were checked and met the standard requirement of NIMH. The two datasets were genotyped using different platforms: the MGS was genotyped using the Affymetrix 6.0 chip that includes 638,937 SNPs, and the batches 5 and 6 of the SSCCS were genotyped using the Illumina OmniExpress chip that includes 646,699 SNPs. In this work, we did not do any SNP annotation as both datasets are using GRCh37/hg19 as the human reference genome. While SSCCS dataset has more subjects, its order of magnitude is approximately the same as of MGS (5334 subjects with 638,937 SNPs in MGS vs. 6731 subjects with 646,699 SNPs in SSCCS) in a sense that we expect both datasets to have similar power in detecting the associations. The genotype of an individual is coded as 0, 1, or 2 whether the subject has 0, 1, or 2 copies of the minor allele. Missing genotypes were imputed by the sample average of the genotypes at the position. Phenotypes were recorded as a binary variable indicating presence or absence of a schizophrenia diagnosis.

### Study design

The objective of the current study is to evaluate whether piMASS can improve the detection of association signals as compared with standard univariate procedure. To this end, we chose a dataset with a moderate sample size that has previously been analyzed using univariate methods.^5^ We did not perform genotype imputation and used the same set of markers as in the study.^5^ Guan and Stephens mention that BVS regression tends to spread the association signal (the PIPs) among the correlated SNPs.^13^ Therefore, to apply piMASS, we partitioned the genome-wide data into smaller regions to capture additive effects of neighboring SNPs. Based on our computational resources, we set 1000 SNPs as the region in a single run. Given that we did not impute genotypes, it was more practical to proceed with regions containing equal number of SNPs. Since piMASS searches for various model configurations by proposing to add, remove, and switch covariates in the model, we used a “sliding window” approach in which each chromosome was cut into regions of 1000 SNPs with the overlap of 500 SNPs. This ensures that piMASS has the opportunity to explore models containing all nearby SNPs. This approach produced 1266 regions in the MGS dataset (the number of regions per chromosome are listed in Supplementary Table 5). A typical region spans around 3–7 million base pairs.

We tested each region for association with phenotype using piMASS by performing Markov chain Monte Carlo (MCMC) runs with one million iterations each. The convergence of the MCMC runs was confirmed by the Gelman–Rubin statistics being <1.04 (Supplementary Notes). We did not include covariates to correct for potential population stratification since it has been noted in the literature that Bayesian regression models simultaneously fitting multiple SNPs are robust for population stratification.^34^ To distinguish SNPs with the strongest evidence of association, we use PIP for each SNP. Since nearby SNPs are usually correlated and the PIPs can spread around correlated SNPs, it is possible that none of the single SNPs in the region have high PIP but the sum of PIPs would be high indicating the posterior probability of at least one of the SNPs should be included in the model. The design of combining multiple loci into a region helps better explore all possible models. Given that the regions are defined in terms of fixed number of SNPs, we used the sum of PIPs as the main measurement of association following analysis.^13^

piMASS allows user to input parameters (priors) appropriate for the specific question in a GWAS and therefore utilize the existing domain knowledge. From the latest GWASs, it is known that more than 100 genetic loci are associated with schizophrenia, but each locus has very small effect.^5–7^ This knowledge can be utilized to specify the ranges of the prior parameters. In the model, we specified the following two parameters: the proportion of the phenotypic variance explained by relevant variants and the proportion of SNPs that we expect to be relevant to the phenotype. The first parameter represents the estimate of overall signal in the genotype data, e.g., how much variation in the diagnosis can be explained by the SNP data. We set a prior on this quantity to be uniformly distributed from 0.01 to 1% according to the two previous schizophrenia studies.^7,35^ These two studies suggest that the variants across the genome collectively explain 18–23% of phenotype variation.^7,35^ But for each variant, the variation explained is very small. Ripke et al.^7^ found 108 variants, the expected proportion explained by a single variant would be 0.184/108 = 0.0017, which falls in the range we used as a priori information in the model. The prior on the second parameter was set in such a way that the expected number of SNPs that are relevant to the phenotype ranges from 1 to 5 loci in each region containing a group of 1000 SNPs. One could also set restriction on the total number of SNPs to be allowed in the model. In this study, we set it to 5 SNPs due to computation time considerations.

The key feature of prior setting in piMASS is that the number of relevant variables is no longer necessarily positively correlated to the proportion of variance explained by them. Such prior structure is proper for the situation where there are many relevant variables, each has tiny effect and overall proportion of variability explained by relevant covariates is still small. This feature of piMASS matches the genetic architecture of schizophrenia where many loci, each with a very small effect, collectively contribute to the disease.

**Fig. 4:**
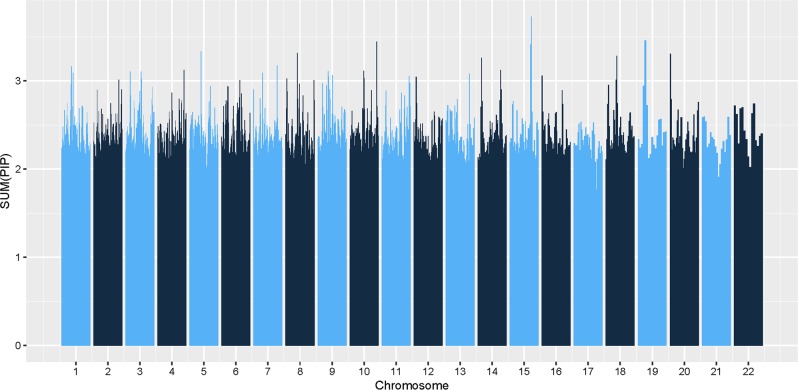
piMASS genome-wide region-based performance of the MGS dataset. The sum of posterior inclusion probabilities (PIPs) for each of the 1266 overlapping regions spanning 22 chromosomes of the MGS dataset

In the initial application of piMASS to the MGS dataset, sum of PIPs for the 1266 regions in the MGS dataset did not show clear separations (Fig. 4). Therefore, it was not clear which regions contain SNPs that are associated with the trait. We borrowed frequentist permutation test to determine an appropriate significance threshold, which would make our results comparable with those reported previously.^5^ We used empirical method based on the Fisher’s concept of a permutation test. Given that 1266 overlapping regions were tested simultaneously, it was necessary to correct the significance threshold for multiple comparisons. We chose Bonferroni correction for this purpose. Since our focus is on gene discovery, we set *α* = 0.1, being more liberal than the traditional 5% level to compensate for the well-known conservativeness of Bonferroni multiple correction. Although the larger *α* may produce more false positives, we expect to eliminate them in the validation step of the study. Each region that survived the significance threshold of $\frac{p}{n} = \frac{{0.1}}{{1266}} \approx 7.9 \times 10^{ - 5}$ was further validated using an independent dataset. The target number of permutations was set to 100,000. To save computation time, we stopped permutations for each region once the region’s empirical *p* value exceeded the significance threshold.

### Validation

The main goal of validation is to check if the results obtained using piMASS could be replicated in an independent dataset so that the method could be applied to discover new loci in general. Several regions in the discovery dataset that performed best in terms of the empirical *p* values from the permutation were tested and verified using an independently collected dataset (SSCCS dataset) with similar sample size. As mentioned above, the two datasets were genotyped using different platforms with different sets of SNPs. As we only included the SNPs that belong to the region with the same position interval, the number of SNPs in each validation region is not exactly 1000. Accordingly, mean of PIPs is used as a measure of association to account for the variable number of markers in the discovery and the corresponding validation regions. We use *α* = 0.05 and correct for multiple comparisons based on the number of regions undergoing the validation. This yields empirical *p* value based on 1000 permutations for each validation region. Since the best performing regions based on the permutation test are in top 2% of all regions according to the initial run using the MGS dataset (Supplementary Table 6), we also conducted the separate piMASS analysis of SSCCS dataset with no permutation test, followed by an overlap analysis of both datasets based on piMASS results without permutation test. In detail, top 5% of the best performing regions based on sum of PIPs were selected. For each such region top 1% of the best performing SNPs based on PIPs were selected, comprising a table of SNPs with the highest PIP among regions with the highest sum of PIPs for each dataset. We checked every SNP in each table if it is within 100,000 bp distance of SNPs from the other dataset. Those SNPs comprise overlap set between the two datasets. Next, for each pair in the overlap set we checked LD between the SNPs in the pair. If two SNPs were in LD, they were considered in the consensus set. The larger size of the consensus set points toward consistency of the piMASS method.

### Reporting Summary

Further information on research design is available in the Nature Research Reporting Summary.

## Supplementary information

Reporting Summary checklist

Supplementary Information

## Data Availability

The molecular genetics of schizophrenia (MGS) data and batches 5 and 6 of the Swedish Schizophrenia Case–Control Study (SSCCS) data that support the findings of this study are available in NIMH Genetic Repository and Resource (https://www.nimhgenetics.org/) upon approval of NIMH.

## Code availability

The code used to generate output has been uploaded to GitHub public repository (https://github.com/rowedata/sczGWAS) under MIT license and includes the description of the input data format.
