# Supplementary material for: Biological and practical implications of genome-wide association study of schizophrenia using Bayesian variable selection
Source: NPJ Schizophr. 2019 Nov 19;5:19. doi: 10.1038/s41537-019-0088-6 (PMC6863898; doi:10.1038/s41537-019-0088-6)
Supplement: Supplementary file 2 — Supplementary Information [file 41537_2019_88_MOESM2_ESM.pdf]

Supplementary Table 1. Top 1% of the SNPs of the best performing regions in permutation test that have been validated

Chr	Region	Start position	End position	P-value from discovery dataset	P-value from validation dataset	Top 1% SNP	SNP location	Known SNP	LD D'	LD R^2	LD p-value	Gene in SZGR 2.0	Gene in GWAS Catalog
15	29	83,907,801	86,887,657	1.43E-05	0.001	rs4887510	84393428						
						rs4887511	84393542						
						rs11856833	84608888						
						rs16977847	84963263						
						rs16940789	86123465					LINC00052, NTRK3	
						rs16941261	86456524					NTRK3	
						rs4887364	86461119					NTRK3	
						rs991728	86463950					NTRK3	
						rs2114252	86465680					NTRK3	
						rs994068	86467650					NTRK3	
19	5	15,724,023	22,638,628	1.67E-04	<0.001	rs17710624	17637294						
						rs6512209	17637331						
						rs7249760	18472545					ELL	
						rs4808135	18476270					ELL (eQTL: eGene: UBA52)	
						rs2965189	19380518	rs2905426, rs2315283	0.9774, 1	0.9299, 0.9519	<0.0001, <0.0001	GATAD2A (eQTL: eGene: ATP13A1, LD)	mapped rs2905426 to GATAD2A and MAU2
						rs2916074	19391270	rs2905426, rs2315283	0.9774, 1	0.9299, 0.9519	<0.0001, <0.0001	GATAD2A (eQTL: eGene: ATP13A1, LD)	
						rs4808200	19415803	rs2905426, rs2315283	0.9955, 1	0.9732, 0.9955	<0.0001, <0.0001	GATAD2A	
						rs4808203	19429659	rs2905426, rs2315283	0.9954, 0.9954	0.9644, 0.982	<0.0001, <0.0001	GATAD2A (eQTL: eGene: ATP13A1, LD)	
						rs10419912	19458055	rs2905426, rs2315283	0.9954, 0.9955	0.9644, 0.9865	<0.0001, <0.0001	GATAD2A (eQTL: eGene: ATP13A1, LD)	
						rs4808964	19464692	rs2905426, rs2315283	0.9954, 0.9955	0.96, 0.9865	<0.0001, <0.0001	GATAD2A (eQTL: eGene: ATP13A1, LD)	

14	33	86,399,092	90,573,122	2.20E-04	<0.001	rs10148955	87845065					KCNK10	
						rs2274736	88008405					PTPN21 (eQTL:eGene: SPATA7)	
						rs2401751	88016375					PTPN21 (eQTL:eGene: SPATA7)	
						rs1864744	88020759					PTPN21 (eQTL:eGene: SPATA7)	
						rs1144918	88172311					EML5	
						rs1287825	88175289					EML5	
						rs1956411	88204113					EML5	
						rs10132509	88273534					EML5	
						rs7154695	89865458						
						rs961196	90204414					TTC7B	
9	24	34,905,605	70,379,322	2.50E-04	0.002	rs10814211	35169278					UNC13B	
						rs1930357	35251744					UNC13B	
						rs10511919	35269814					UNC13B	
						rs1930358	35302364					UNC13B	
						rs10972434	35319331					UNC13B	
						rs1927959	35346685					UNC13B	
						rs7855134	70264671						
						rs265077	70336353						
						rs7861495	70344427					TMEM252	
						rs11789244	70346200						
8	30	53,113,091	57,376,926	3.00E-04	0.001	rs1871094	54641512						
						rs2169692	54642493						
						rs2086074	54646328						
						rs7842388	54662041						
						rs13266723	54667270						
						rs12547226	54668493						
						rs16921164	56198701					XKR4	
						rs16921167	56199413					XKR4	
						rs9643832	56211316						
						rs868541	57039314						

14	6	29,288,170	33,177,081	3.00E-04	<0.001	rs8016004	31407748					NUBPL, ARHGAP5-AS1 Func: intergenic	
						rs9322878	31411657					NUBPL, ARHGAP5-AS1 Func: intergenic	
						rs6571481	31424827					NUBPL, ARHGAP5-AS1 Func: intergenic	
						rs1953271	31434298						
						rs2378971	31439957						
						rs915071	31503609	rs2068012	in equilibrium			NUBPL, ARHGAP5-AS1 Func: intergenic	Mapped gene(s): LOC105370439, LOC105370440, Traits: mental or behavioral disorder, reported traits: bipolar disorder and schizophrenia
						rs972396	31505063					NUBPL, ARHGAP5-AS1 Func: intergenic	
						rs1952966	31508235					NUBPL, ARHGAP5-AS1 Func: intergenic	
						rs1953439	32542174						
						rs1609496	32931905						
20	1	9,795	2,715,620	3.33E-04	<0.001	rs6135141	22347						
						rs910389	842722					ANGPT4	
						rs6132408	2120408						
						rs6137288	2120546						
						rs6082408	2140516						
						rs6082421	2145908						
						rs6137352	2146288						
						rs6082426	2146635						
						rs6047529	2163286						
						rs6515654	2547121					TMC2	
18	15	28,642,588	33,646,071	5.00E-04	<0.001	rs4799699	29320850						
						rs8084529	29779207					NOL4	
						rs7238808	29793737					NOL4	
						rs4799754	30146408						
						rs7240718	30610808						
						rs9966287	30616548						
						rs894775	33124749					CELF4	

						rs7241842	33126739					CELF4	
						rs4799910	33129733					CELF4	
						rs1893455	33130790					CELF4	
15	28	80,260,648	85,190,202	5.50E-04	<0.001	rs17359001	81328522					HOMER2	
						rs2562769	82026934					SH3GL3	
						rs2880974	84216204						
						rs1444295	84392851					KLHL25, LINC01584 intergenic	
						rs1444296	84392875					KLHL25, LINC01584 intergenic	
						rs4887510	84393428						
						rs4887511	84393542						
						rs4887425	84607834						
						rs11856833	84608888						
						rs16977847	84963263	rs950169	in equilibrium				
1	36	81,955,643	85,727,849	5.67E-04	<0.001	rs6675025	83468598						
						rs17101527	83482778						
						rs17101590	83515305						
						rs11163719	83565239						
						rs3864596	83568538						
						rs6704084	83807136					LINC01361, LOC101927587, intergenic	
						rs12033001	83810786					LINC01361, LOC101927587, intergenic	
						rs11163772	83816880					LOC101927587, ncRNA_exonic	
						rs4140462	84041887						
						rs12065881	84992456					SSX2IP, LPAR3, intergenic	
13	41	98,395,342	#########	6.00E-04	<0.001	rs17610319	98907781						
						rs17610347	98912364						
						rs3751403	100499748					NALCN-AS1, ncRNA_intronic	
						rs12430088	100502077					NALCN-AS1, ncRNA_intronic	

						rs2044117	100506311					NALCN-AS1, ncRNA_intronic	Mapped gene(s): NALCN, NALCN-AS1, Traits: mental or behavioral disorder, Reported traits: Bipolar disorder and schizophrenia
						rs638732	100507599					NALCN-AS1, ncRNA_exonic	
						rs9554752	100524314					NALCN	Mapped gene(s): NALCN, Trait(s): bipolar disorder, eating disorder; Reported traits: bipolar disorder and eating disorder
						rs7986657	100535000					NALCN	
						rs875117	100973881						
						rs875116	100973899						
3	15	22,010,347	25,354,138	7.50E-04	0.001	rs7613236	24644441						
						rs2362758	24644963						
						rs4858630	24699887						
						rs4858631	24700007						
						rs4302350	25026121						
						rs4280597	25028486						
						rs1603993	25052792					MIR4792, RARB, intergenic	
						rs9847186	25056861					MIR4792, RARB, intergenic	
						rs993804	25087175					MIR4792, RARB, intergenic	Mapped gene(s): RARB, Traits: mental or behavioral disorder; Reported traits: Bipolar disorder and schizophrenia
						rs4858697	25091586						Mapped gene(s): RARB, Trait(s): body mass index, physical activity measurement; reported traits: Body mass index, Body mass index (joint analysis main effects and physical activity interaction), Body mass index in physically active individuals

Supplementary Table 2. Overlap set of SNPs with a consensus set

Chr	rsID (MGS)	Location	PIP	rsID (SSCCS)	Location	PIP	LD D’	LD R^2	LD p-value
2	rs12692173	236135601	0.05678	rs13020554	236214007	0.56308	in equil.		
2	rs12692173	236135601	0.05678	rs1864713	236228570	0.43186	in equil.		
2	rs6718421	236119282	0.02946	rs13020554	236214007	0.56308	in equil.		
6	rs12530421	29657422	0.06324	rs2747421	29645118	0.33439	0.7717	0.1451	<0.0001
6	rs12530421	29657422	0.06324	rs2535238	29645038	0.21314	0.7717	0.1451	<0.0001
6	rs12530421	29657422	0.06324	rs375984	29644502	0.13229	0.7717	0.1451	<0.0001
6	rs12530421	29657422	0.06324	rs29218	29607429	0.11607	0.7557	0.1628	<0.0001
6	rs7746199	27369303	0.05953	rs9379982	27376267	0.33668	in equil.		
6	rs7746199	27369303	0.05953	rs7750290	27385481	0.09477	in equil.		
6	rs1883213	26411157	0.04472	rs7755741	26337264	0.11807	0.9814	0.8925	<0.0001
6	rs1883213	26411157	0.04472	rs6915468	26410577	0.18509	in equil.		
6	rs3025645	29675600	0.03077	rs2747421	29645118	0.33439	0.8103	0.1537	<0.0001
6	rs3025645	29675600	0.03077	rs2535238	29645038	0.21314	0.8103	0.1537	<0.0001
6	rs3025645	29675600	0.03077	rs375984	29644502	0.13229	0.8103	0.1537	<0.0001
6	rs3025645	29675600	0.03077	rs29218	29607429	0.11607	0.7925	0.172	<0.0001
6	rs994379	26413386	0.02957	rs7755741	26337264	0.11807	0.9892	0.6133	<0.0001
6	rs994379	26413386	0.02957	rs6915468	26410577	0.18509			
6	rs29255	29687523	0.02884	rs2747421	29645118	0.33439	0.9196	0.174	<0.0001
6	rs29255	29687523	0.02884	rs2535238	29645038	0.21314	0.9196	0.174	<0.0001
6	rs29255	29687523	0.02884	rs375984	29644502	0.13229	0.9196	0.174	<0.0001
6	rs29255	29687523	0.02884	rs29218	29607429	0.11607	0.9218	0.2046	<0.0001
6	rs2267633	29678820	0.02835	rs2747421	29645118	0.33439	in equil.		
6	rs2267633	29678820	0.02835	rs2535238	29645038	0.21314	in equil.		
6	rs2267633	29678820	0.02835	rs375984	29644502	0.13229	in equil.		
6	rs2267633	29678820	0.02835	rs29218	29607429	0.11607	in equil.		
11	rs1245133	112516931	0.33551	rs1459658	112565534	0.13176	in equil.		
11	rs1273044	112499058	0.10368	rs1459658	112565534	0.13176	in equil.		
11	rs11214533	112553676	0.05071	rs1459658	112565534	0.13176	in equil.		
11	rs6589360	112555502	0.04858	rs1459658	112565534	0.13176	in equil.		
11	rs12272966	112539997	0.04746	rs1459658	112565534	0.13176	in equil.		
11	rs10502170	112545328	0.03535	rs1459658	112565534	0.13176	in equil.		
14	rs1609496	32931905	0.56483	rs8015624	32897944	0.51485	in equil.		
16	rs3747600	4526223	0.04612	rs6500602	4497451	0.35187	0.841	0.6818	<0.0001
16	rs860891	4526530	0.04229	rs6500602	4497451	0.35187	0.792	0.5307	<0.0001
16	rs2160567	4475558	0.03735	rs9938257	4382607	0.09494	0.6479	0.2992	<0.0001
16	rs2160567	4475558	0.03735	rs6500602	4497451	0.35187	0.9	0.7397	<0.0001
16	rs2302553	4996296	0.03472	rs12445265	4924160	0.42264	in equil.		
16	rs2302553	4996296	0.03472	rs2734742	4938160	0.10818	in equil.		
16	rs2302553	4996296	0.03472	rs12445265	4924160	0.13271	in equil.		
16	rs2302553	4996296	0.03472	rs966062	4907430	0.11048	in equil.		
16	rs2270366	4498616	0.03116	rs6500602	4497451	0.35187	0.8996	0.7291	<0.0001

Supplementary Table 3. Cutoff distance and degree of overlap between MGS and SSCCS dataset

Distance (bp)	Number of SNPs in overlap	% of SNPs in overlap out of 640 SNPs
10,000	4	0.6%
50,000	27	4.2%
100,000	41	6.4%
300,000	112	17.5%
500,000	202	31.6%
1,000,000	405	63.3%

Supplementary Table 4. Overlap between the consensus set and PGC results

PGC rsID	Chr	Dataset	rsID	LD D	LD R^2	LD p-value
rs115329265	6	SSCCS	rs2747421	0.4485	0.1537	<0.0001
rs115329265	6	SSCCS	rs2535238	0.4485	0.1537	<0.0001
rs115329265	6	SSCCS	rs375984	0.4485	0.1537	<0.0001
rs115329265	6	SSCCS	rs2747421	0.4485	0.1537	<0.0001
rs115329265	6	SSCCS	rs2535238	0.4485	0.1537	<0.0001
rs115329265	6	SSCCS	rs375984	0.4485	0.1537	<0.0001
rs115329265	6	MGS	rs7746199	0.635	0.3479	<0.0001

Supplementary Table 5. Number of regions per chromosome for MGS and SSCCS dataset

Chromosome	Number of regions (MGS)	Number of regions (SSCCS)
1	103	105
2	107	102
3	89	84
4	80	71
5	83	74
6	84	85
7	70	67
8	72	66
9	61	59
10	71	70
11	65	65
12	63	63
13	49	48
14	41	41
15	38	39
16	39	41
17	30	36
18	38	38
19	17	26
20	33	27
21	18	18
22	15	19

Supplementary Table 6. Best performing regions based on permutation test and their genome wide rank according to the initial run using the MGS dataset

Region	Chromosome	Permutation p-value	Genome wide rank
9	24	0.0000	24
15	29	0.0000	1
19	5	0.0000	2
8	30	0.0001	6
14	33	0.0001	12
14	6	0.0002	9
5	34	0.0003	5
4	74	0.0004	13
20	1	0.0004	7
13	41	0.0004	20
15	28	0.0004	4
7	58	0.0004	10
9	23	0.0005	15
9	32	0.0005	21
18	15	0.0005	8
1	36	0.0007	11
3	15	0.0008	16

Supplementary Notes. Convergence diagnostics for piMASS with binary response data

Gelman–Rubin diagnostics

For the convergence analysis we used 2 separate 1 million step runs with different starting values, sampling every 10 steps. This yielded 2 chains with 100,000 observations each that are used for convergence analysis. The variable we are analyzing is an estimate of heritability based on sampling posterior distribution.

Summary of the first chain:

Iterations = 1,000,000
Sample size per chain = 100,000

1. Empirical mean, standard deviation, and standard error of the mean:

Mean	SD	Naive SE	Time Series SE
4.340e-03	2.958e-03	9.353e-06	1.510e-04

2. Quantiles:

2.5%	25%	50%	75%	97.5%
0.000	0.002	0.004	0.006	0.011

Figure 1. Trace plot and density plot for chain 1

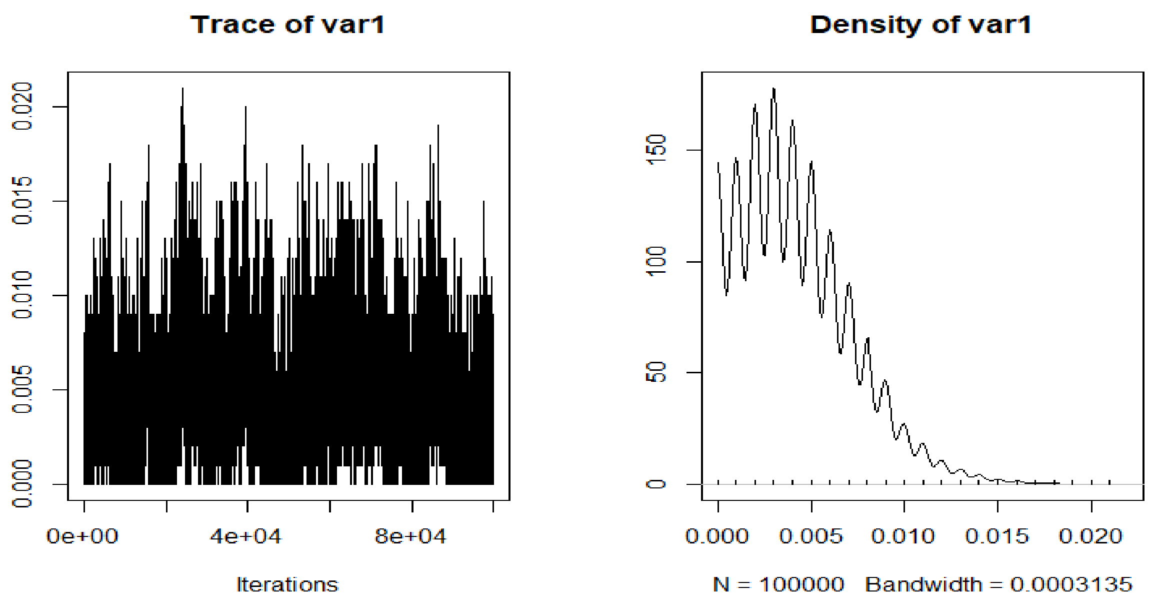

Summary of the second chain:

Iterations = 1,000,000

Sample size per chain = 100,000

1. Empirical mean, standard deviation, and standard error of the mean:

Mean	SD	Naive SE	Time Series SE
4.165e-03	3.086e-03	9.760e-06	1.748e-04

2. Quantiles:

2.5%	25%	50%	75%	97.5%
0.000	0.002	0.004	0.006	0.012

Figure 2. Trace plot and density plot for chain 2

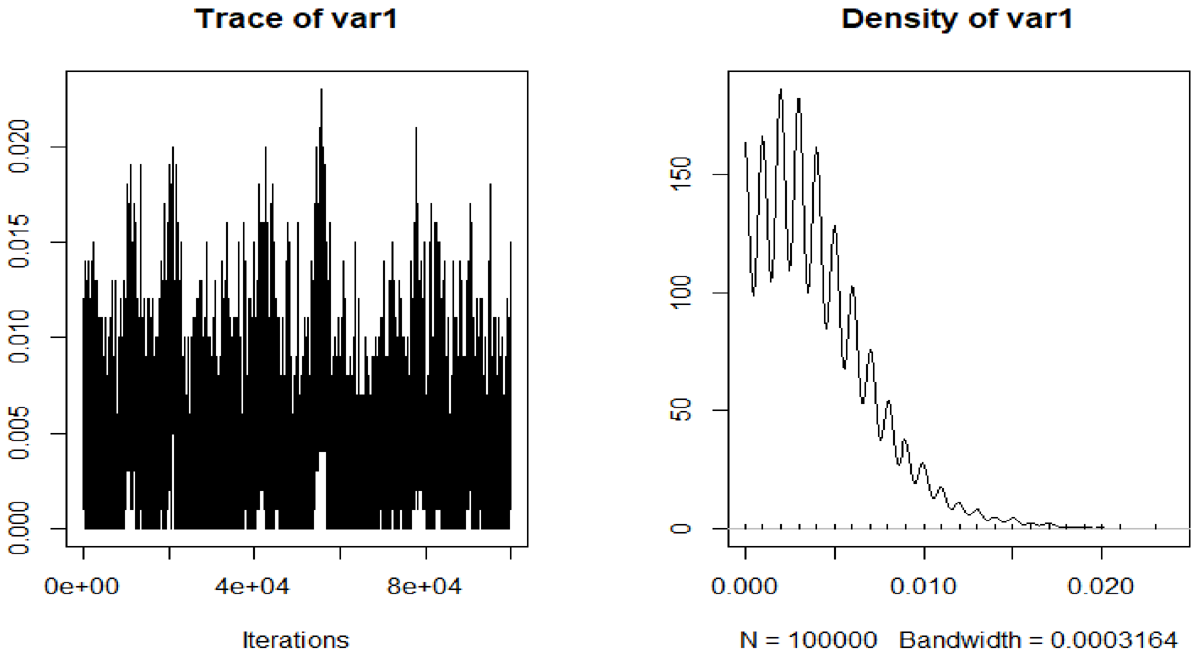

We combined two chains and calculated Gelman-Rubin scale reduction factor. The trace and density plots for both chains combined are displayed below:

Figure 3. Trace plot and density plot for combined chains

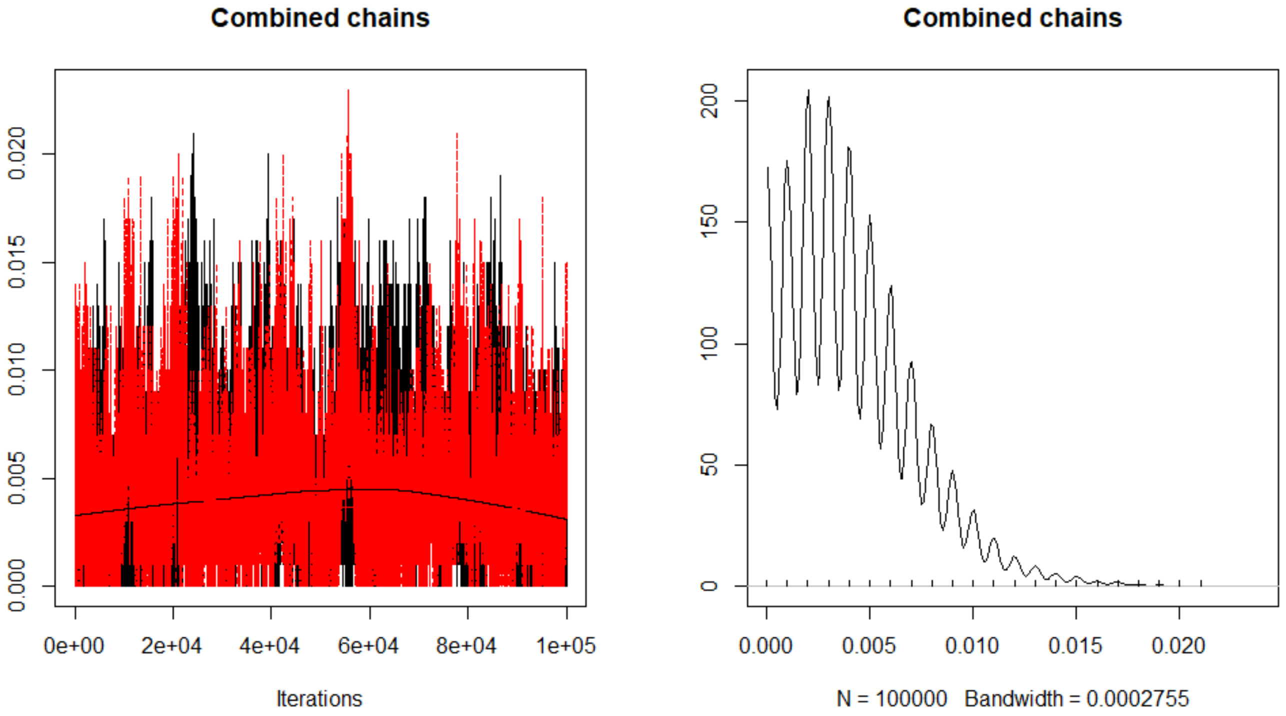

Point estimate of potential scale reduction factor is 1.01 with upper 95% CI of 1.04.

Figure 4. Scale reduction factor for 2 chains over time

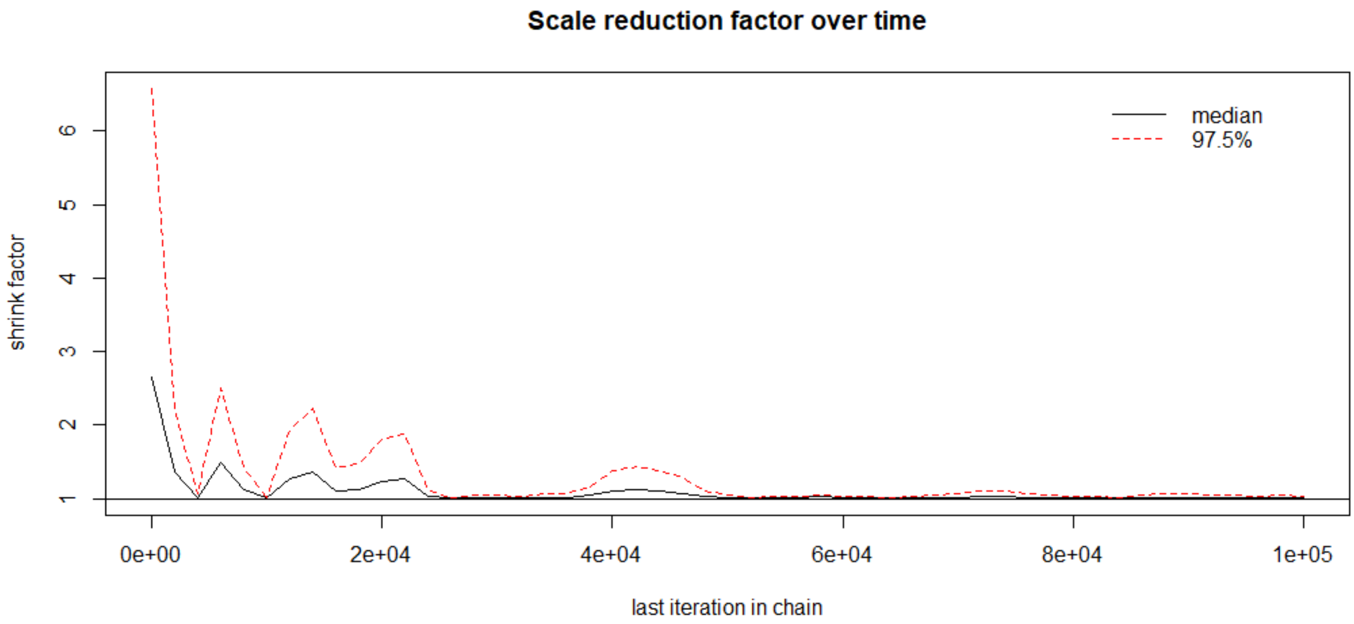

The scale reduction plot shows the development of the scale-reduction over time (chain steps). The plot is also a tool to see roughly where the point from which the chains seem converged. On the graph above after 50,000 iterations chains look to be converging.

Table 1. Summary statistics for 1 million steps run split into 10 groups

	Mean	Median	Standard deviation
1	0.0037	0.003	0.0027
2	0.0032	0.003	0.0026
3	0.0055	0.005	0.0034
4	0.0051	0.005	0.0031
5	0.0037	0.003	0.0026
6	0.0044	0.004	0.0027
7	0.0056	0.005	0.0029
8	0.0048	0.004	0.0031
9	0.0046	0.004	0.0027
10	0.0028	0.002	0.0021
Total	0.0043	0.004	0.0030

Table 2. Summary statistics for 100 million steps run split into 10 groups

	Mean	Median	Standard deviation
1	0.0042	0.004	0.0029
2	0.0044	0.004	0.0031
3	0.0043	0.004	0.0029
4	0.0041	0.004	0.0029
5	0.0041	0.004	0.0028
6	0.0043	0.004	0.0030
7	0.0043	0.004	0.0030
8	0.0042	0.004	0.0029
9	0.0042	0.004	0.0029
10	0.0043	0.004	0.0030
Total	0.0042	0.004	0.0029

Below are plots showing 1 million and 100 million step chains split into 10 groups each.

Figure 5. Trace plots below provide show million iterations run yielding 100,000 recorded observations split into 10 groups

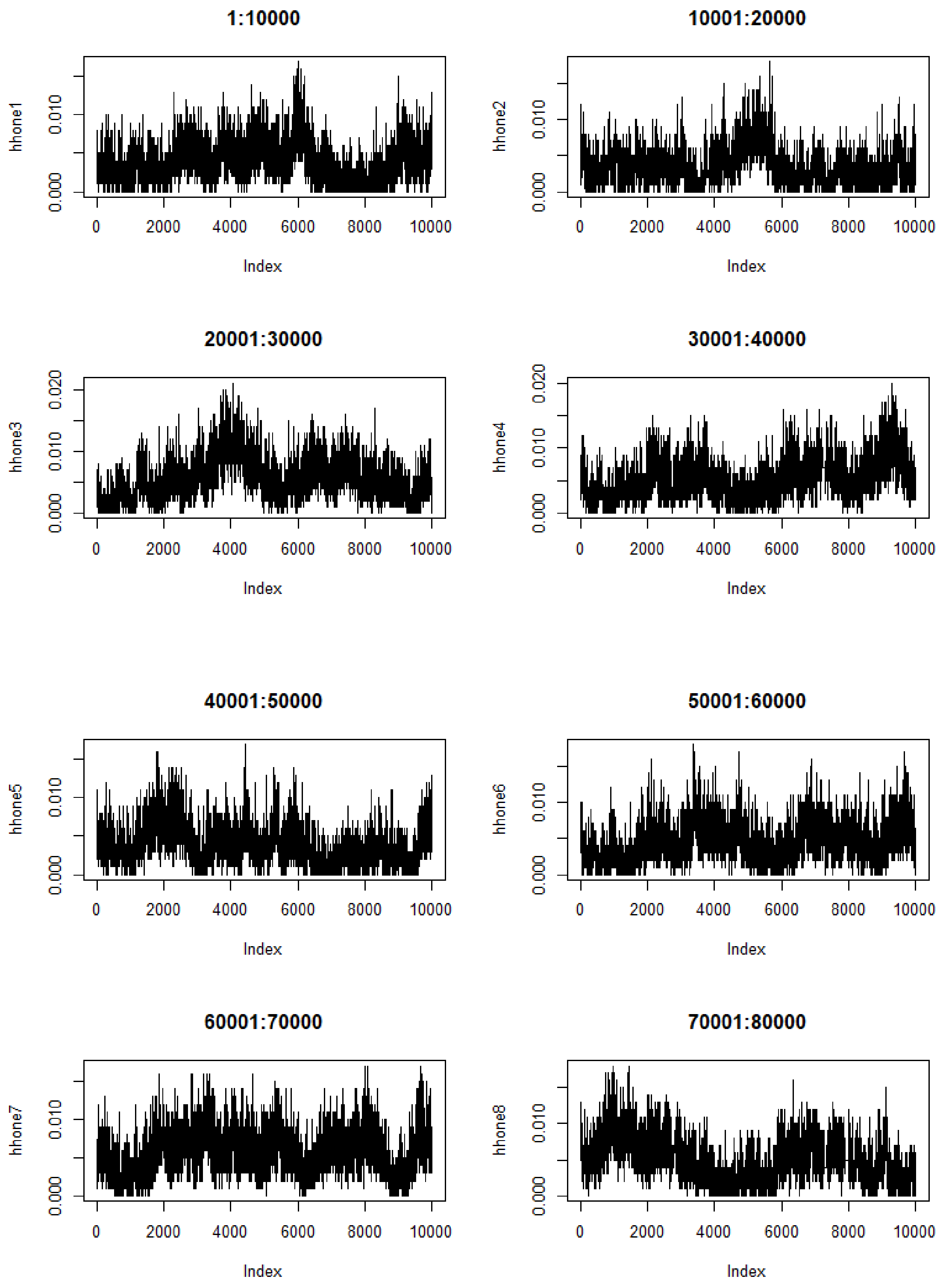

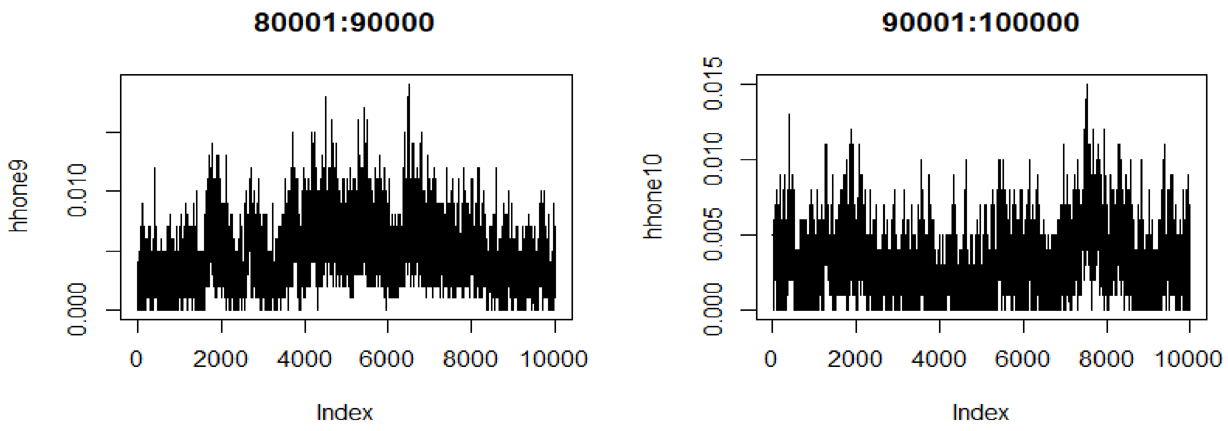

Figure 6. Trace and density plots of the full run

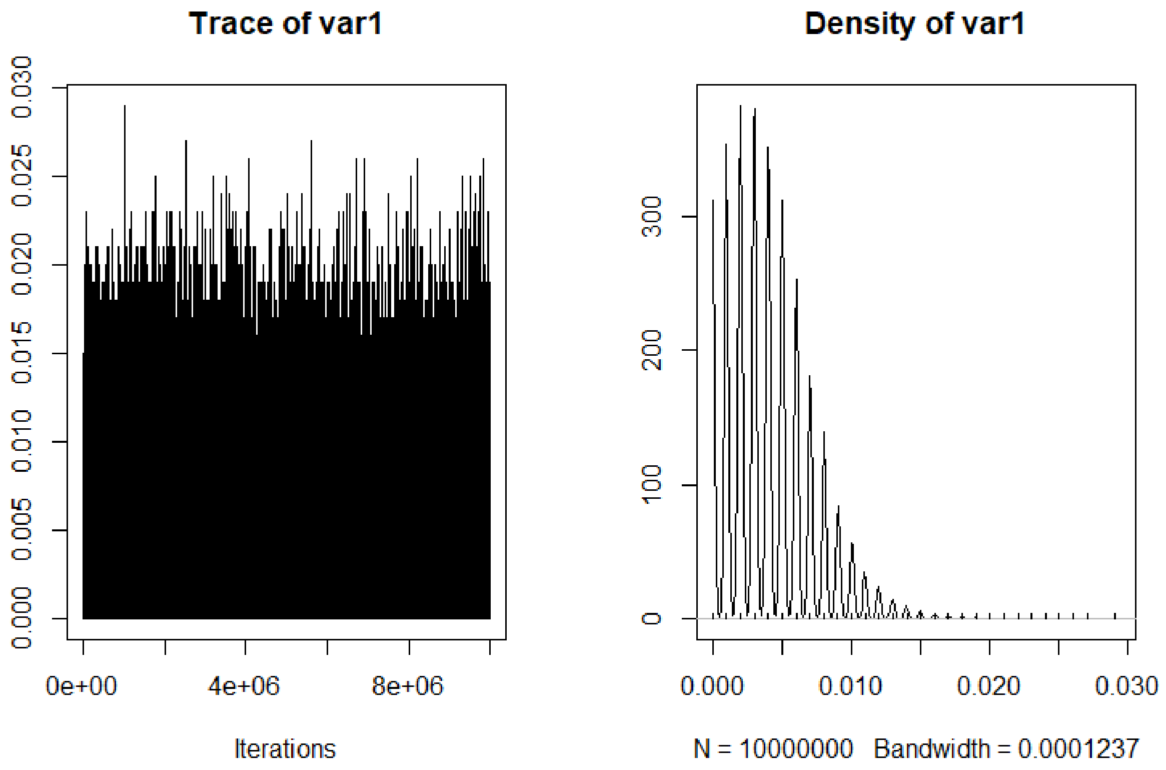

Figure 7. Hundred million iterations run yielding 10,000,000 recorded observations split into 10 groups

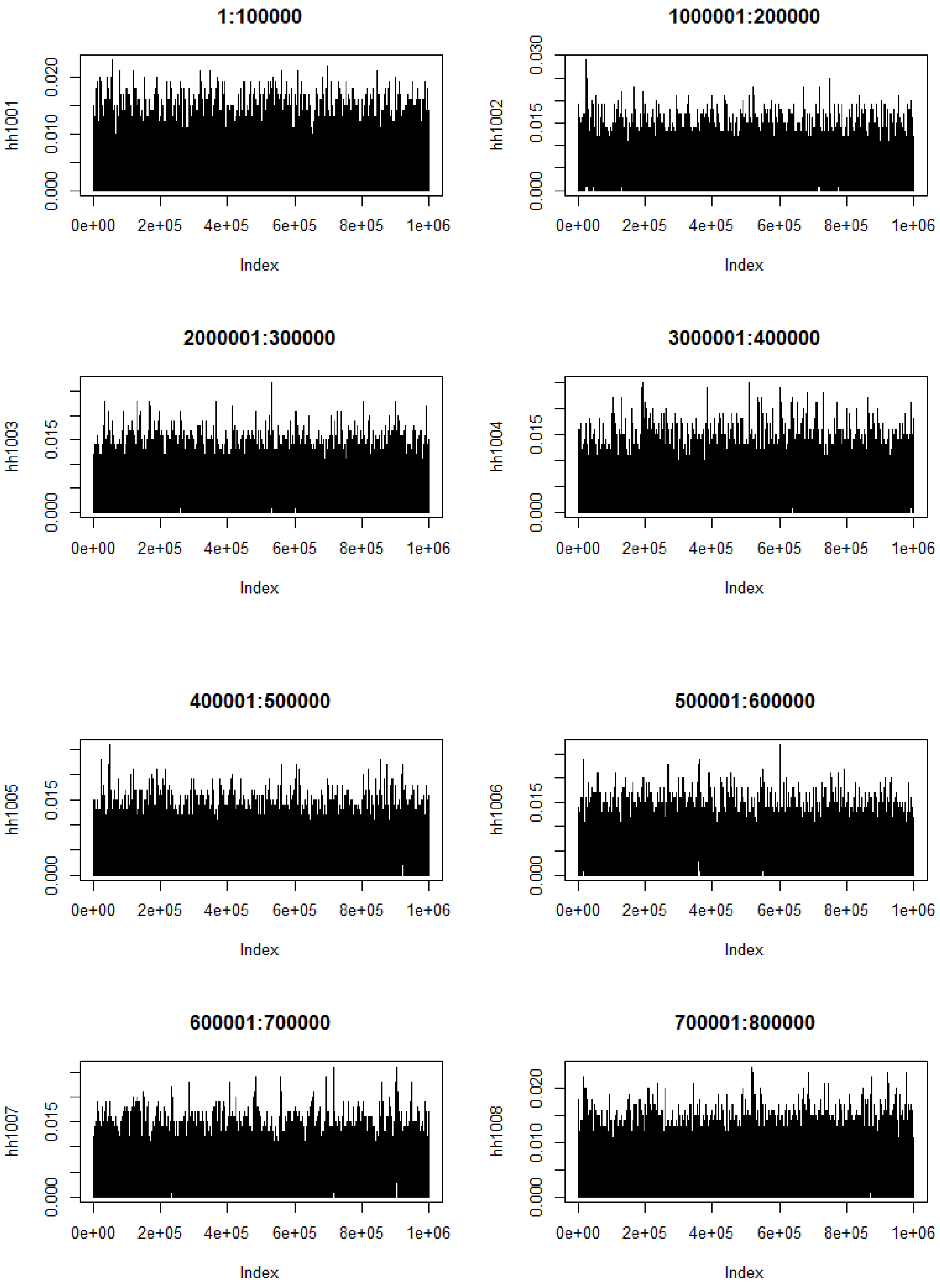

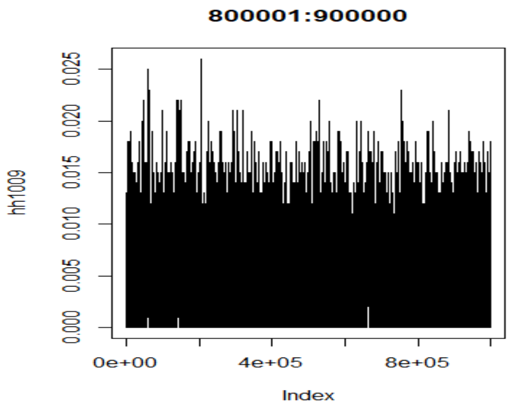
800001:900000
hh1009
0.000
0.005
0.010
0.015
0.020
0.025
0e+00
4e+05
8e+05
Index

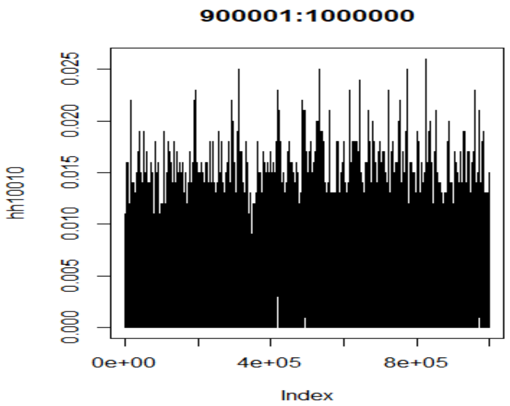
900001:1000000
hh10010
0.000
0.005
0.010
0.015
0.020
0.025
0e+00
4e+05
8e+05
Index
